# Raman tensor of zinc-phosphide (Zn_3_P_2_): from polarization measurements to simulation of Raman spectra†

**DOI:** 10.1039/d1cp04322f

**Published:** 2021-11-24

**Authors:** Mischa Flór, Elias Z. Stutz, Santhanu P. Ramanandan, Mahdi Zamani, Rajrupa Paul, Jean-Baptiste Leran, Alexander P. Litvinchuk, Anna Fontcuberta i Morral, Mirjana Dimitrievska

**Affiliations:** Laboratory of Semiconductor Materials, Institute of Materials, Faculty of Engineering, Ecole Polytechnique Fédérale de Lausanne 1015 Lausanne Switzerland mirjana.dimitrievska@epfl.ch; Texas Center for Superconductivity and Department of Physics, University of Houston Houston Texas 77204-5002 USA; Institute of Physics, Faculty of Basic Sciences, Ecole Polytechnique Fédérale de Lausanne 1015 Lausanne Switzerland

## Abstract

Zinc phosphide (Zn_3_P_2_) is a II–V compound semiconductor with promising photovoltaic and thermoelectric applications. Its complex structure is susceptible to facile defect formation, which plays a key role in further optimization of the material. Raman spectroscopy can be effectively used for defect characterization. However, the Raman tensor of Zn_3_P_2_, which determines the intensity of Raman peaks and anisotropy of inelastic light scattering, is still unknown. In this paper, we use angle-resolved polarization Raman measurements on stoichiometric monocrystalline Zn_3_P_2_ thin films to obtain the Raman tensor of Zn_3_P_2_. This has allowed determination of the Raman tensor elements characteristic for the A_1g_, B_1g_ and B_2g_ vibrational modes. These results have been compared with the theoretically obtained Raman tensor elements and simulated Raman spectra from the lattice-dynamics calculations using first-principles force constants. Excellent agreement is found between the experimental and simulated Raman spectra of Zn_3_P_2_ for various polarization configurations, providing a platform for future characterization of the defects in this material.

## Abstract 2

Raman tensor of zinc phosphide (Zn_3_P_2_) is determined using angle-resolved polarization Raman measurements and first principles calculations. These results provide a platform for future characterization of the defects in this material.

## Graphical abstract

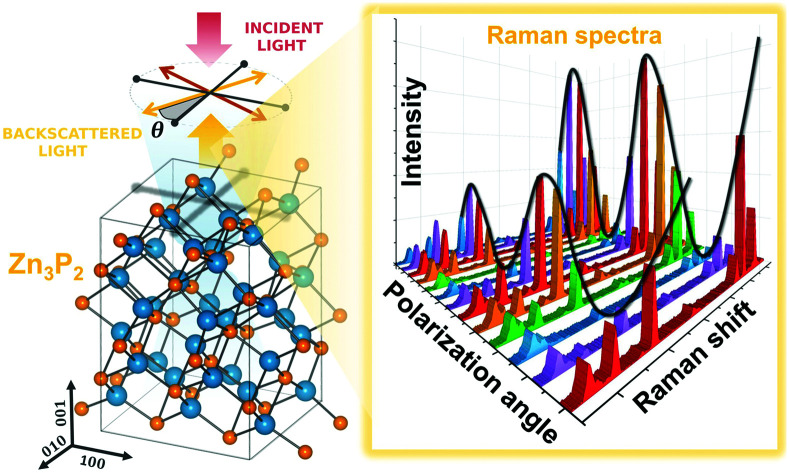

## 1 Introduction

Zinc Phosphide (Zn_3_P_2_) is a semiconductor made of earth-abundant elements, with promising properties for photovoltaic and thermoelectric applications.^1–14^ Recently, Zn_3_P_2_ has also been receiving more attention due to its facile synthesis into nanostructures,^15–18^ as well as breakthroughs in growth quality.^19–21^ One attractive feature of Zn_3_P_2_ is a large and complex tetragonally distorted fluorite lattice, which ensures phase stability over a substantial range of compositions, thanks to the disseminated vacancies.^22,23^ This offers tunability for defect engineering and doping, a stepping stone to achieving efficient Zn_3_P_2_-based devices.^24–26^ An important element to advancing the defect engineering in Zn_3_P_2_ and related compounds, is developing a fast and non-destructive method for defect characterization and quantification. ^25,26^

Raman spectroscopy is a versatile tool for studying structural changes in materials, as it provides information about the vibrational properties of the material both due to the crystal structure and chemical composition. Considering that phonons represent collective vibrational oscillations of atoms inside the lattice, any kind of structural irregularities, such as defects or impurities, will strongly affect them. Thus, Raman spectroscopy may serve as a powerful technique for providing information on phase,^27^ defects,^28–31^ inhomogeneities,^32^ and crystallinity.^33^

In order to successfully use Raman spectroscopy for identification of defects, it is necessary to build methodologies which combine both experimental and computational tools. A common challenge in experimental studies is distinguishing the exact type and quantity of defects present. This leads to difficulties in establishing correlation between certain types of defects and changes in the Raman features. Computational tools could help in this case, by simulating Raman spectra of materials with various types and concentrations of defects. However, these simulations are usually challenging, as they require building very large supercells, which result in high computational costs. Recently, a new method for simulating Raman spectra of defective materials with a small computational cost was proposed by Hashemi *et al.*^34^ These simulations use vibrational eigenvectors of the defective system and the Raman tensors of the pristine system to calculate the Raman spectra of a defective material. Therefore, the first step towards application of Raman spectroscopy for defect identification in Zn_3_P_2_ is reliable determination of its Raman tensor elements on the reference samples.

Previous work on Raman characterization of Zn_3_P_2_ was mostly aimed at providing reference Raman spectra. First elucidations on the properties of Raman modes were proposed from group theory analysis,^22^ following by experimental investigations,^23,35^ and more recently from first principle calculations based on density functional theory (DFT).^35^ Stutz *et al.*^35^ have used DFT calculations and Raman polarization measurements on a single crystal Zn_3_P_2_ nanowires, allowing identification of 33 phonon modes which were attributed to their respective symmetries. To the best of our knowledge, Raman tensor elements of Zn_3_P_2_ have not yet been investigated or reported, even though they are essential for establishing tangible correlations between Raman mode intensities and the existence of defects.^36^ Additionally, Raman spectra simulations of Zn_3_P_2_ have been challenging to perform due to the large number of atoms involved in the Zn_3_P_2_ crystal lattice.

In this work, we provide new insights on the vibrational properties of stoichiometric monocrystalline Zn_3_P_2_ thin films using angle-resolved polarization Raman measurements and first-principle calculations. Particular focus is put on the intensity evolution of the Raman modes under different polarization configurations. This has allowed determination of the Raman tensor elements characteristic for the A_1g_, B_1g_ and B_2g_ vibrational modes. These results have been compared with the theoretically obtained Raman tensor elements and simulated Raman spectra from the lattice-dynamics calculations.

## 2 Experimental methods

### Sample preparation

Monocrystalline Zn_3_P_2_ thin films were grown by molecular-beam epitaxy on InP(100) substrates. The growth methods are extensively explained in reference^19^.

### Polarized Raman spectroscopy

Polarized Raman spectroscopy was realized in the backscattering configuration at 12 K. The 488 nm line of a Coherent sapphire optically pumped semiconductor laser was used for excitation. The beam was focused on the sample with a microscope objective with a numerical aperture of 0.75, resulting in a 1 μm diameter spot, reaching a radiant power of the order of 700 μW. The incident flux was controlled combining a half-waveplate and a polarization beam-splitter. The incident polarization was controlled with a subsequent half-waveplate. The backscattered light goes through a linear polarizer, and a last half-waveplate directs the polarization parallel to the entrance slit of a TriVista triple spectrometer with 900, 900 and 1800 cm^−1^ gratings in subtractive mode and a Princeton Instrument liquid nitrogen cooled multichannel CCD PyLoN camera. The polarization direction is described with respect to a reference direction on the setup and moves in the *xy* crystallographic plane of the sample. All the spectra were calibrated based on the reference sulfur Raman spectrum.

### Structural and compositional characterization

High angle annular dark field (HAADF) STEM image and energy dispersive X-ray spectroscopy (EDS) elemental maps were collected using FEI Talos transmission electron microscope operating at 200 kV.

### Density functional theory calculations

The first-principles calculations of the electronic ground state of the tetragonally structured Zn_3_P_2_ were performed within the local density approximation using Ceperly–Adler functional,^37,38^ as implemented in the CASTEP code.^39^ Norm-conserving pseudopotentials were used. The cutoff energy for the plane wave basis was set to 600 eV. A selfconsistent-field (SCF) tolerance better than 10^−7^ eV per atom and the phonon SCF threshold of 10^−12^ eV per atom were imposed. Prior to performing calculations, the structure was relaxed so that forces on atoms in the equilibrium position did not exceed 2 meV Å^−1^ and the residual stress was below 5 MPa. Experimentally determined lattice parameters from ref. 40 were used as a starting point. An integration over the Brillouin zone was performed over a 3 × 3 × 2 Monkhorst–Pack grid in reciprocal space.

## 3 Results and discussion

### 3.1 Structural and compositional characterization of Zn_3_P_2_ thin film

**Fig. 1:**
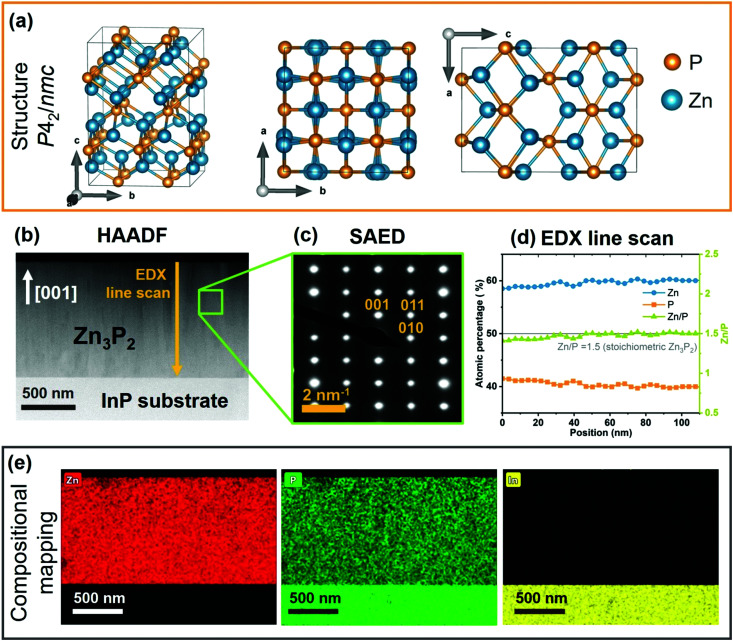
Structural and compositional characterization of Zn_3_P_2_ thin film: (a) Crystal structure representation of tetragonal Zn_3_P_2_ unit cell along different crystal planes. (b) HAADF image of the Zn_3_P_2_ thin film cross section along with (c) the SAED pattern confirming its monocrystalline nature. The patterns are indexed with respect to the tetragonal (*P*4_2_/*nmc*) unit cell. (d) EDX line scan and (e) compositional mapping of the Zn_3_P_2_ thin film showing uniform distribution of Zn and P along the cross section, without any In intermixing from the InP(100) substrate. The measured composition of the Zn_3_P_2_ thin film is Zn/P = 1.51 ± 0.02.

We start by providing evidence of the crystal structure and composition of the sample investigated here. Fig. 1(a) presents the crystal structure of Zn_3_P_2_ by visualizing it from three different zone axes. The lattice has a tetragonal symmetry with *P*4_2_/*nmc* (*D*^15^_4h_) space group. The unit cell contains 8 formula units (Zn_24_P_16_) with 40 atoms total, and lattice parameters of *a* = 8.0785 Å and *c* = 11.3966 Å.^40^ Cations (Zn) and anions (P) are stacked in alternate planes along the *c*-axis. The unit cell can be described as an assembly of four cubic sub-cells similar to the fluoride structure, except for one quarter of the cations missing. These are Zn vacancies located on the body diagonals of the cubes. The irregular ordering of these vacancies results in distortions that lowers the symmetry, which explains the four times larger volume compared to fluoride-like unit cells. Zn cations form distorted tetrahedrons of which the vertices are the P anions. The P anions are surrounded by 8 voids of which 6 are occupied by the Zn cations.

Fig. 1(b) dispatches the cross sectional HAADF image of the synthesized Zn_3_P_2_ thin film on InP(100) substrate. Selective area electron diffraction (SAED) pattern (Fig. 1(c)) measured on the Zn_3_P_2_ layer confirms the monocrystalline nature of the sample. Calculations of atomic distances based on the SAED pattern corroborate the formation of the tetragonal (*P*4_2_/*nmc*) unit cell of Zn_3_P_2_ and the growth direction along the *c* crystal axis ([001]), which is perpendicular to the substrate. The compositional assessment, performed by STEM-EDX (Fig. 1(d)), indicates formation of close to stochiometric phase with Zn/P = 1.51 ± 0.02 (compared to stochiometric Zn/P = 1.5). Further, a homogenous distribution of Zn and P with no detectable phase segregation or intermixing with indium (In) is observed from the compositional maps shown in Fig. 1(e). This is within the spatial and chemical composition resolution of our experiments and agrees well with previous studies.^19^

### 3.2 Raman tensors of Zn_3_P_2_

Group theory analysis predicts 39 Raman active modes for Zn_3_P_2_ structure with the following irreducible representations:^41^

1 Γ_Raman_ = 9A_1g_ + 10B_1g_ + 4B_2g_ + 16E_g_

where the A and B modes correspond to non-degenerate modes, while the E modes are doubly degenerate.

Under non-resonant conditions, the Raman tensors for the Raman-active phonon modes are defined as:2
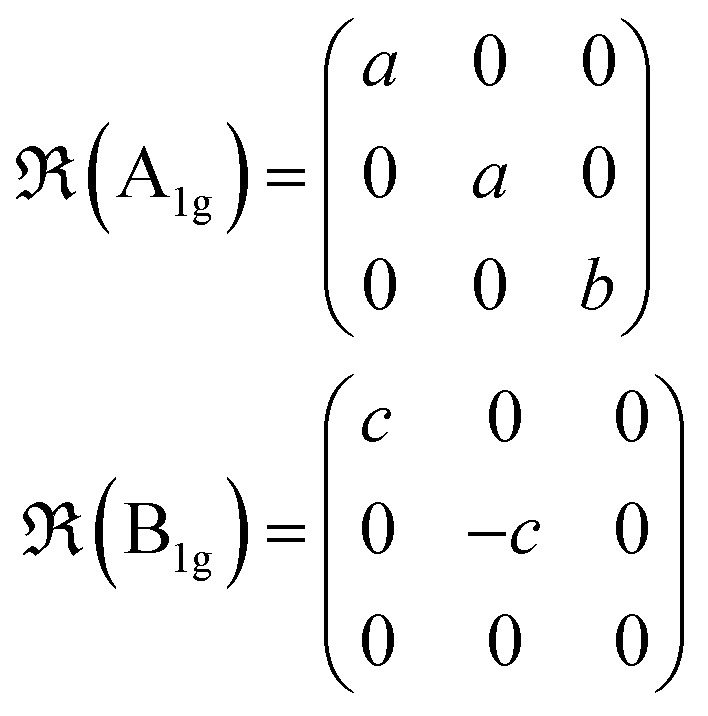

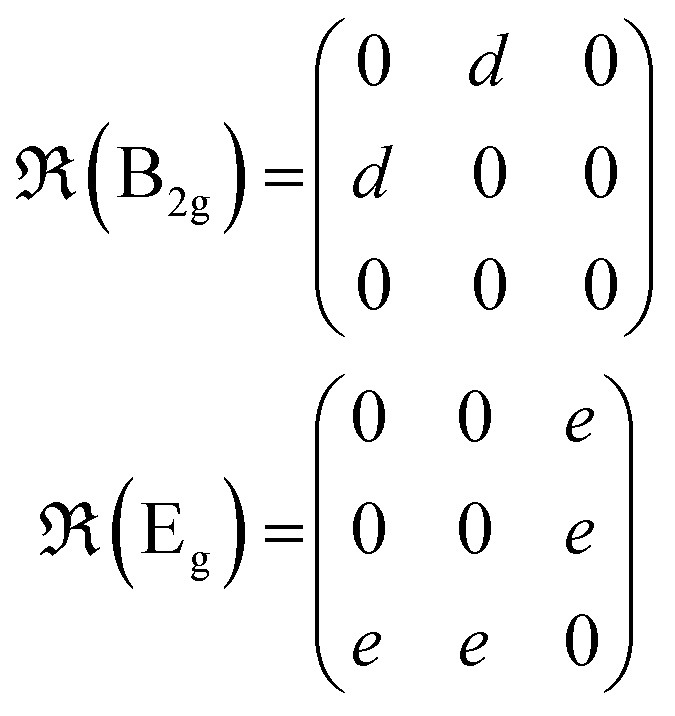
where *a*, *b*, *c*, *d* and *e* are the Raman tensor coefficients (elements).

The scattering intensity of each mode in the Raman spectra is defined by:^42^3
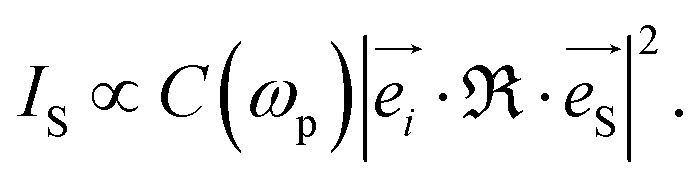
where 
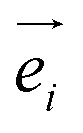
 and 
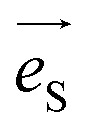
 are the unitary polarization vectors of the incident light and backscattered light, respectively. The coefficient *C*(*ω*_p_), while usually omitted in the literature, should be considered here, as it describes the dependence of the Raman mode intensity from the phonon frequency *ω*_p_ and the incident laser frequency *ω*_*i*_. It is defined as:^42^4
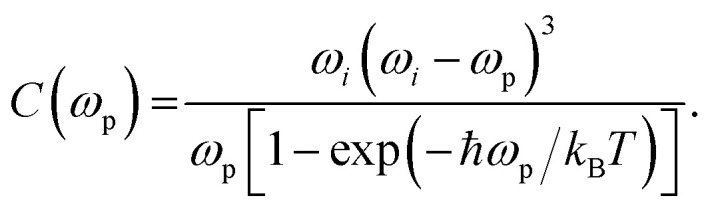
where *ħ* = *h*/2π, with *h* being the Planck constant, *k*_B_ is the Boltzmann constant and *T* is the temperature of the measurements.

**Fig. 2:**
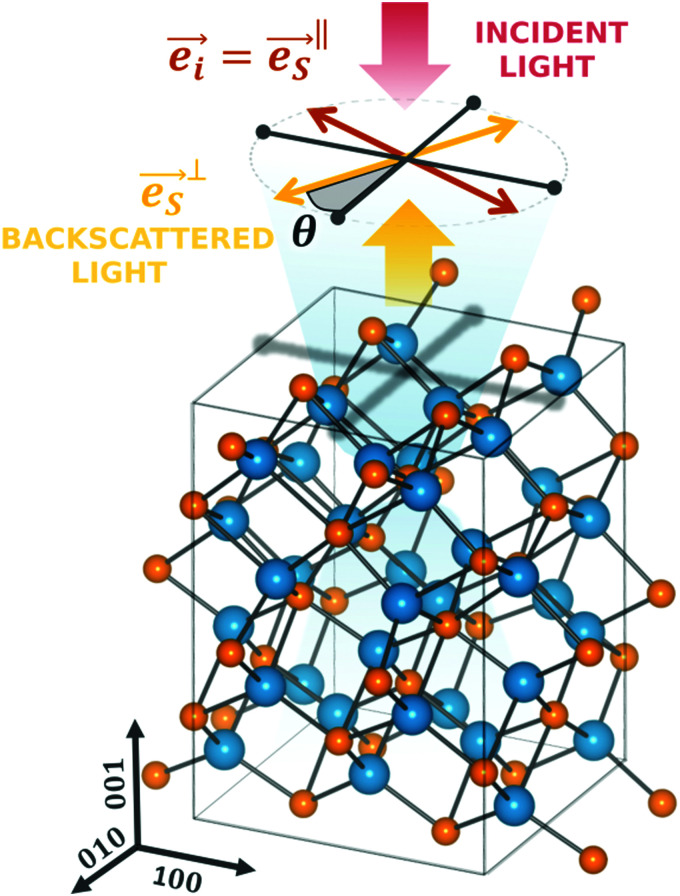
Schematic of the Raman scattering polarization measurements. Raman measurements are performed on (001) basal plane with incident laser light (wide red arrow) projected along the *z*-axis ([001] direction). The incident light polarization and the output polarization in perpendicular configuration are represented by the thin red and yellow arrows, respectively. The black arrows indicate the *x* and *y* axis of the crystal corresponding to [100] and [010] directions, respectively.

Considering the configuration of the Raman measurements as shown in Fig. 2, the incident 
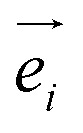
 and backscattering 
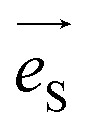
 polarization unit vectors can be defined as:5
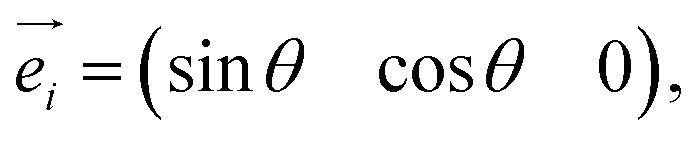
6
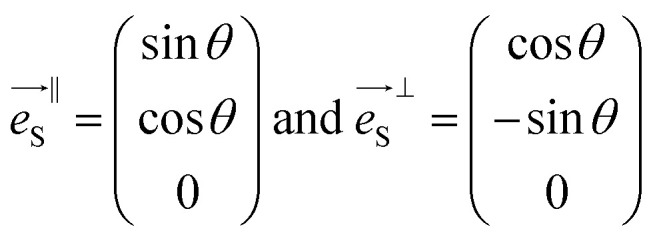
where *θ* is the polarization angle with respect to the [010] axis, while the signs ‖ and ⊥ correspond to either parallel or perpendicular polarization, respectively.

Combination of eqn (3)–(6) yields the Raman intensity dependency from the polarization angle for different phonon modes, which are presented in Table 1. We find that changes in the polarization angle lead to selective activation or cancelling of phonon modes. In the case where polarization Raman measurements are performed incident onto the (001) basal plane (Fig. 2), the E_g_ modes are silent while the other modes vary according to the polarization configuration. The intensity of the B_1g_ and B_2g_ modes should periodically change in phase opposition of π/4 (45°), while the intensity of the A_1g_ mode is constant in parallel configuration and cancelled in perpendicular.

**Table 1:** Angular dependencies of Raman modes intensity for tetragonal Zn_3_P_2_, in parallel and perpendicular measurement configurations on (001) basal plane, with *θ* being the polarization angle between 
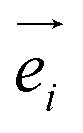
 and (010)

	Parallel configuration	Perpendicular configuration
A_1g_ phonon modes	*C*(*ω*_p_)·*a*^2^	0
B_1g_ phonon modes	*C*(*ω*_p_)·|*c*·cos 2*θ*|^2^	*C*(*ω*_p_)·|*c*·sin 2*θ*|^2^
B_2g_ phonon modes	*C*(*ω*_p_)·|*d*·sin 2*θ*|^2^	*C*(*ω*_p_)·|*d*·cos 2*θ*|^2^
E_g_ phonon modes	0	0

### 3.3 Angle-resolved Raman polarization measurements of Zn_3_P_2_

**Fig. 3:**
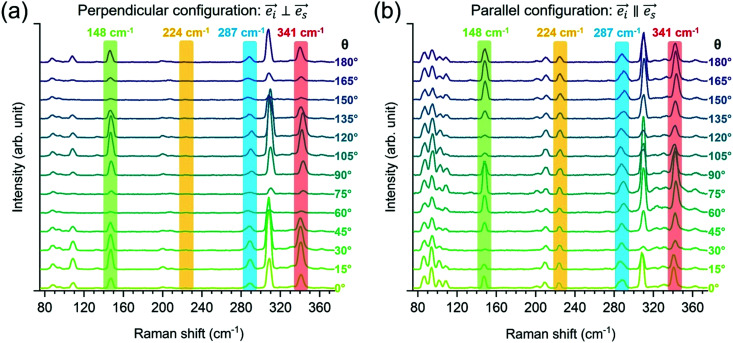
Polarization Raman spectra of monocrystalline Zn_3_P_2_ thin film measured for various polarization angles (*θ*) in (a) perpendicular and (b) parallel configuration. The spectra were acquired at 12 K and using 488 nm excitation wavelength. Both configurations were measured under the same experimental conditions. For legibility the spectra are shifted vertically by a constant value. Raman peaks at 148, 224, 287 and 341 cm^−1^ are highlighted as representative peaks with characteristic polarization intensity behavior.

We move now to the experimental Raman spectroscopy measurements. Fig. 3 presents the polarization Raman spectra performed on monocrystalline Zn_3_P_2_ thin film, in parallel and perpendicular configurations for various polarization angles (*θ*) in the range from 0 to 180°. All spectra were acquired at low temperature (12 K) to reduce thermal effects on the Raman peaks, leading to increased intensity and reduced peak-broadening due to increased phonon lifetime at lower temperatures. We chose 488 nm laser to avoid resonance Raman effects, as based on the band structure calculations for Zn_3_P_2_,^14^ there are no electronic bands in the energy region corresponding to the laser excitation. Detailed analysis of the complete set of Raman spectra allowed resolution of 17 peaks which intensity evolves according to the expected behavior in Table 1, enabling attribution of each peak to A_1g_, B_1g_ or B_2g_ mode as further elucidated in the next paragraphs.

**Fig. 4:**
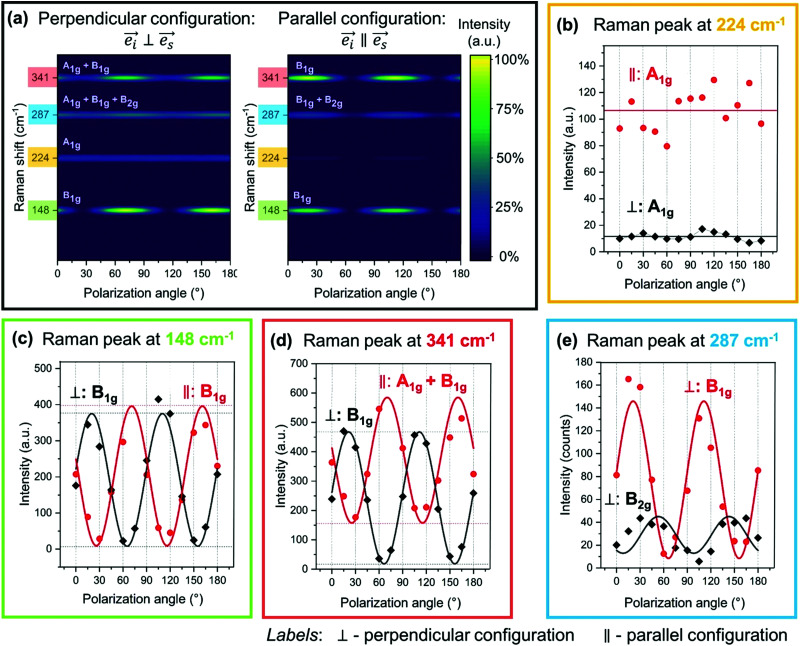
(a) Calculated Raman intensity profiles in dependence of polarization angle in perpendicular and parallel configurations for four representative Raman peaks. (b–e) Polarization-dependent Raman intensities for four characteristic Raman peaks centered at 224, 148, 341 and 287 cm^−1^, showing the modeling of the experimental data according to the equations presented in Table 1 and allowing calculation of the Raman tensor elements. Dot and diamond symbols represent the experimental data, while full lines present the fits. The standard uncertainty in the intensity (represented by dots and diamonds in the figure) is considered to be ±5%.

We highlight four representative Raman peaks centered at 148, 224, 287 and 341 cm^−1^ with characteristic polarization intensity behavior in Fig. 3. Clearly, the intensity of the highlighted peaks varies with *θ* for peaks at 148 and 341 cm^−1^, while these changes are less obvious for peaks at 224 and 287 cm^−1^. This behavior can be visualized in a more accurate manner by plotting the intensity as a function of *θ* as in Fig. 4(a). Fig. 4(a) illustrates very clearly which peaks exhibit either a periodic change with *θ* or no variation at all. Fig. 4(b–e) plot the intensity values with respect to *θ*, allowing for a fit of the curves. Based on the fittings, it is possible to unambiguously identify the symmetry type of each peak observed in the Raman spectra of Zn_3_P_2_. Raman peaks centered at 95 cm^−1^ and 224 cm^−1^ exhibit a constant intensity in parallel configuration and cancelled in perpendicular configuration (Fig. 4(b)). This is a typical behavior of A_1g_ modes, as pointed out in Table 1. In contrast, the intensity of Raman peaks at 109 cm^−1^, 148 cm^−1^, 201 cm^−1^, and 309 cm^−1^ evolve sinusoidally in both configurations, as shown in Fig. 4(c). This is consistent with the behavior of B_1g_ modes.

Interestingly, some angular dependencies of peak intensities show a significant intensity offset between the parallel and perpendicular configurations in addition to their sinusoidal evolution. This is the case of the Raman peak at 341 cm^−1^, as shown in Fig. 4(d). This attests an A_1g_ mode superimposed to the present B_1g_ or B_2g_ modes. Hence the peaks observed at 87 cm^−1^, 330 cm^−1^, and 341 cm^−1^ are resulting from A_1g_ and B_1g_ contributions, while the peak at 210 cm^−1^ arises from a superposition of the A_1g_ and a B_2g_ phonon modes.

Finally, the peak around 287 cm^−1^ has the most complex intensity behavior. Observed variation in this peak position with polarization angles (Fig. S1 in the ESI†), as well as wider peak width when compared to other peaks in the Raman spectra, indicate superposition of two or more vibrational modes in this region. Perpendicular configuration offers easer resolution of this peak, considering that the A_1g_ modes are cancelled in this case. Based on the overall intensity dependence with the polarization angle for this peak, it is reasonable to assume that it consists of one B_1g_ and one B_2g_ mode. Having in mind the phase opposition in the intensity behavior between B_1g_ and B_2g_ modes for different polarization angles, these two phonons cannot be simultaneously cancelled or fully activated. This means that for certain polarization angles *θ* only B_1g_ mode will be activated, while B_2g_ mode will be canceled. On the other hand, the situation is reversed for polarization angle *θ* + π/4, where B_2g_ mode is activated, while B_1g_ is canceled. Raman spectra measured under such polarization conditions are presented in Fig. S2(a) in the ESI.† Deconvolution of the Raman spectra measured under these conditions, with Lorentzian curves, has allowed the exact determination of the positions for the weak B_2g_ mode centered at 287 cm^−1^ and the B_1g_ mode centered at 290 cm^−1^. Fig. S2(b) in the ESI† presents an example of the deconvolution under polarization angles for which both B_1g_ or B_2g_ mode are activated. Fig. 4(e) shows the angle resolved intensity dependence of the B_1g_ and B_2g_ modes obtained from the 287 cm^−1^ peak deconvolution for each polarization angle in perpendicular configuration. This observed intensity dependence agrees well with the expected behavior for both B_1g_ and B_2g_ modes as presented in Table 1.

**Fig. 5:**
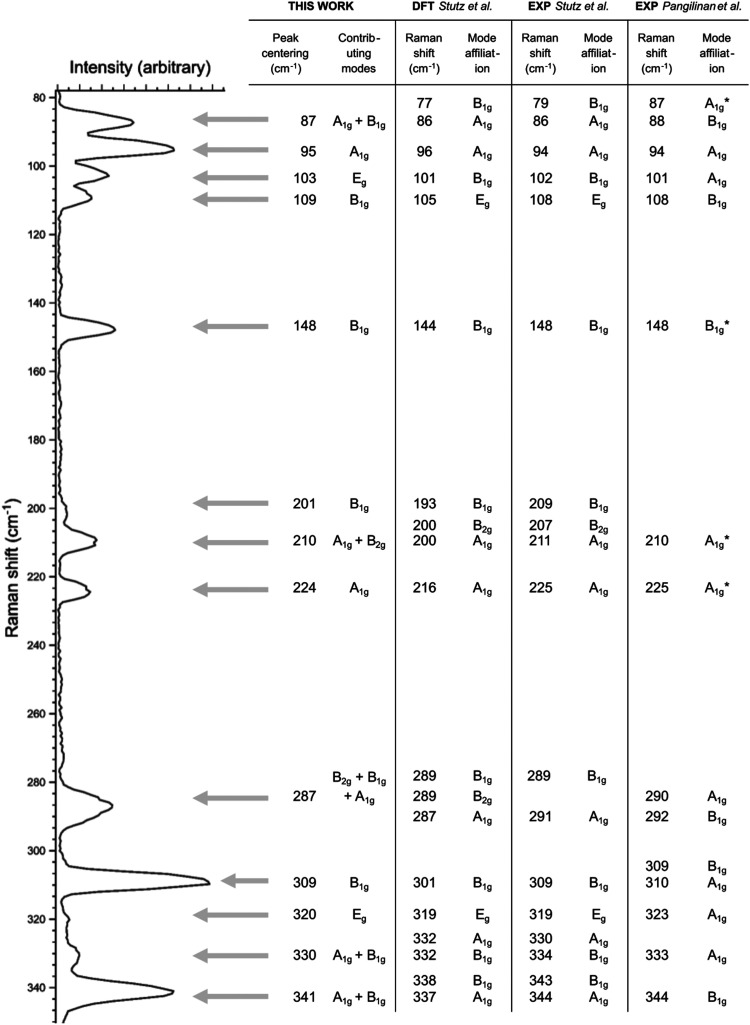
Raman spectrum of monocrystalline Zn_3_P_2_ thin film measured in parallel configuration 
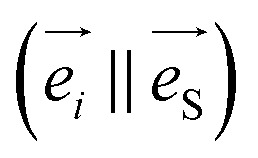
 for polarization angle of *θ* = 22.5°, allowing activation of B_1g_, B_2g_ and A_1g_ modes. Each Raman peak is identified with a position and symmetry assignment as indicated in the table under “This work” column. These are compared with previously reported data based on DFT simulations (column “DFT Stutz *et al.*”^35^) and experiments (columns “EXP Stutz *et al.*”^35^ and “EXP Pangilinan *et al.*”^23^). Asterix symbol (*) in the column “EXP Pangilinan *et al.*” denotes an affiliation to acceptor defect states identified in ref. 23.

The above-mentioned methodology has been applied to all measured Raman spectra and has allowed identification of positions and the angle resolved intensity profiles of 17 Raman active modes in the case of the monocrystalline Zn_3_P_2_ with (001) growth orientation perpendicular to the substrate (Fig. 3). The positions of these modes and their symmetry assignment are listed in Fig. 5, along with the comparison to ref. 23 and 35. Most of the observed Raman peaks are perfectly consistent with those reported in ref. 35. However, two peaks behave unexpectedly. The peak at 103 cm^−1^ has a periodic intensity dependence from polarization angle in both parallel and perpendicular configuration which does not correspond to either B_1g_ or B_2g_ mode (Fig. S3 in the ESI†). This is in contrast to DFT calculations which predict a B_1g_ mode at this position. Furthermore, the intensity evolution of the peak at 109 cm^−1^ suggests an assignment to B_1g_ mode (Fig. S4 in the ESI†), in contrast to an E_g_ mode reported at this position in ref. 35. Considering that the two peak frequencies are close, it is most likely that the Raman peak at 109 cm^−1^ should be assigned to the B_1g_ mode, while the peak at 103 cm^−1^ may in fact correspond to an activated E_g_ mode or a multi-phonon contribution. Considering that the width of the peak at 103 cm^−1^ is similar to the widths of the other peaks in the Raman spectra belonging to one-phonon modes, it is most likely that the multi-phonon contribution could be discarded. Even though E_g_ modes are silent under the used polarization configurations, geometrical reasons can lead to their accidental activation,^43^ such as a slightly titled sample or outer rays from the microscope that result in a vertical component of the incident polarization vector. This same reason may explain the unexpected weak Raman contribution at 320 cm^−1^, corresponding to an activated E_g_ mode, as reported in ref. 35, with a similar intensity evolution as the peak at 103 cm^−1^ (Fig. S5 in the ESI†).

### 3.4 Raman tensor coefficients

Raman tensor elements were calculated for each mode based on the fittings of the Raman intensity dependence with the polarization angle by using equations presented in Table 1. Representative examples of fittings are presented in Fig. 4(b–e). The obtained coefficients are summarized in Table 2. Coefficients *c* have been calculated twice from both perpendicular and parallel polarization configurations and show to be in good agreement. All Raman tensor elements are normalized to the coefficient of the B_1g_ mode at 309 cm^−1^ in perpendicular configuration. It should be noted that Raman tensor element *b* corresponding to the A_1g_ mode and element *e* corresponding to the E_g_ modes could not be calculated in this case, due to the selection rules which apply for the Raman excitation on the (001)-plane basal plane of Zn_3_P_2_.

**Table 2:** Raman tensor coefficients obtained from the fit parameters of the angular intensity dependencies of the phonon modes for Zn_3_P_2_. All elements are normalized to the Raman tensor coefficient of the B_1g_ mode at 309 cm^−1^ in perpendicular configuration. Labels: *ν*_R_ (cm^−1^) – Raman mode frequency; ‖ – parallel polarization configuration; ⊥ – perpendicular polarization configuration

A_1g_ modes		B_1g_ modes			B_2g_ modes	
*ν*_R_ (cm^−1^)	Coefficient *a*	*ν*_R_ (cm^−1^)	Coefficient *c* (‖)	Coefficient *c* (⊥)	*ν*_R_ (cm^−1^)	Coefficient *d*
86	7.1 ± 0.9	79	4.9 ± 1.7	3.8 ± 0.6	207	2.5 ± 0.7
95	8.5 ± 1.1	109	5.4 ± 0.6	6.7 ± 1.0	287	1.5 ± 0.6
210	3.2 ± 0.4	148	9.2 ± 1.1	9.0 ± 0.6		
224	3.7 ± 0.6	201	2.4 ± 0.3	2.6 ± 0.2		
287	3.3 ± 0.6	290		3.9 ± 0.4		
330	2.0 ± 0.5	309	9.6 ± 1.2	10.0 ± 0.4		
343	3.6 ± 1.2	334	2.0 ± 0.5	2.3 ± 0.3		
		341	6.3 ± 0.9	6.4 ± 0.2		

The uncertainties on the tensor elements were estimated using the student's *t*-distribution based on the fitting standard deviation, assuming that the experimental points are normally distributed around the theoretical curve.^44^ The uncertainty generated by the peak centering, that feeds through the tensor elements because of the *C*(*ω*_p_) factor, is negligible and thus not considered. The reflection-induced ellipticity is negligible in our setup, and the spectrum baseline has been subtracted individually following the photoluminescence spectrum line shape, thus avoiding interferences with the baseline angular dependency.

### 3.5 Raman spectra simulations of Zn_3_P_2_ and comparison with the experiments

Raman tensor element calculations for Zn_3_P_2_ were performed in order to compare them with the experimental values. The values were calculated from the first-order dielectric tensor for the equilibrium crystal configuration and for the crystal with atomic displacement according to the vibrational patterns of the individual phonon modes. Raman intensities were then calculated according to equations given in Table 1 and using theoretical values of the Raman tensor elements coupled with the experimental conditions (polarization configuration, laser wavelength and temperature) in agreement with our Raman measurements.

**Fig. 6:**
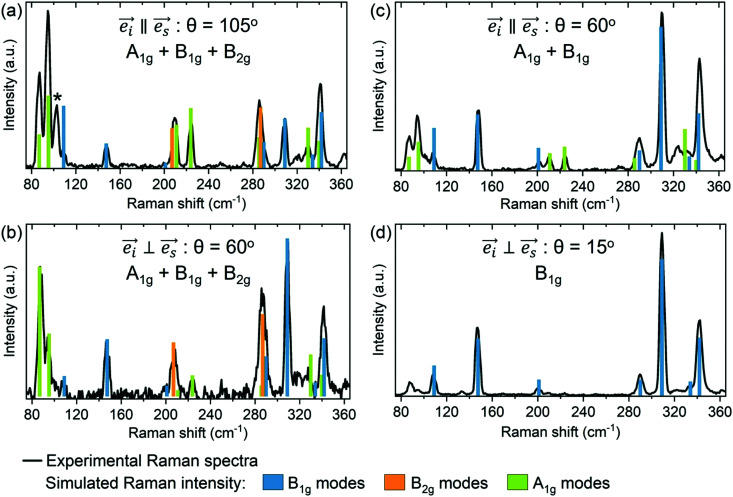
Comparison between the calculated intensity of the Raman modes (colored bars) and the experimental Raman spectra (black line) for four different polarization configurations allowing activation of different phonon modes: (a) parallel configuration 
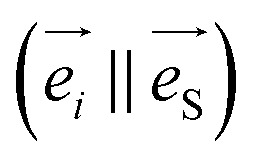
 with polarization angle of *θ* = 105°, allowing activation of B_1g_, B_2g_ and A_1g_ modes; (b) perpendicular configuration 
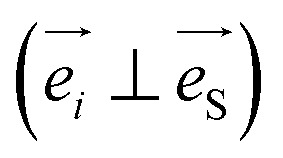
 with polarization angle of *θ*= 60°, allowing activation of B_1g_, B_2g_ and A_1g_ modes; (c) parallel configuration 
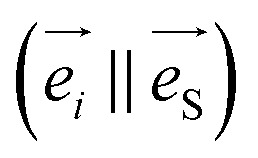
 with polarization angle of *θ* = 60°, allowing activation of B_1g_ and A_1g_ modes; and (d) perpendicular configuration 
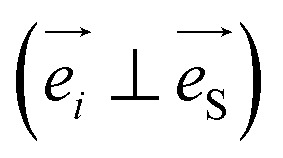
 with polarization angle of *θ* = 15°, allowing activation of B_1g_ modes. Asterix symbol (*) denotes the position of the activated E_g_ mode.

Fig. 6 presents the comparison between the calculated intensities of the Raman modes and the experimental Raman spectra for four polarization configurations, including parallel 
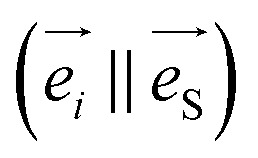
 and perpendicular 
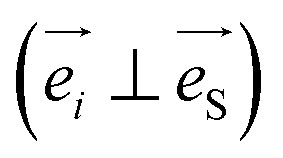
 geometries with various polarization angles (*θ*). These configurations allow activation of different types of phonon modes, such as B_1g_, B_2g_ and A_1g_ in case of Fig. 6(a and b), B_1g_ and A_1g_ in case of Fig. 6(c) and B_1g_ in case of Fig. 6(d). The variety of polarization configurations serves as reliable way for comparison between the experimental and theoretical results.

Overall, Fig. 6 suggests a very good agreement between the theory and experiment. In particular, calculated intensities of the B_1g_ and B_2g_ modes match very well the measured Raman spectra in the high frequency region (>150 cm^−1^). In contrast, there is slightly more discrepancy between the experimental and calculated values in Raman intensities of these modes in the lower frequency region (<150 cm^−1^). Additionally, it is noticed that calculated intensities of the A_1g_ modes are usually over estimated in the high frequency region, and under estimated in the low frequency region. There are several possible reasons for these kinds of discrepancies. First reason is related to the way Raman intensities are calculated in DFT, where certain approximations are necessary for making feasible calculations. These include approximations in the many body interactions, which can become especially important for structures with large number of atoms, such as Zn_3_P_2_. Other possible sources of mismatch in the simulations include the use of a structure optimized at 0 K, as well as a periodic crystal with no treatment of defects or disorder. Defects especially can affect the intensities of Raman modes.^30,31^ Considering that Zn_3_P_2_ is intrinsically p-type semiconductor, due to the low energy formation of P interstitials,^26^ it is possible that these intrinsic defects are affecting the intensities of certain modes in the Raman spectra, and thus creating discrepancy between the experimental and theoretical results. Pangilinan *et al.* has suggested several A_1g_ and B_1g_ modes which could be related to acceptor states in the Raman spectrum of Zn_3_P_2_.^23^ Considering that literature reports on defects structure in Zn_3_P_2_ are limited, further experimental and theoretical explorations on Zn_3_P_2_ defect states are planned in the future for better optimization of the material for optoelectronic applications.

## Conclusion

Vibrational properties of stoichiometric monocrystalline Zn_3_P_2_ were reported from angle-resolved polarization Raman measurements and first-principles calculations. Particular focus was put on the intensity evolution of the Raman modes under different polarization configurations. This has allowed determination of the Raman tensor elements characteristic for A_1g_, B_1g_ and B_2g_ vibrational modes. Simulation of the Raman peak intensities from first-principles calculations allowed direct comparison between the experimental and theoretical values of the Raman tensor elements. Overall good agreement is found between the experimental and simulated Raman spectra of Zn_3_P_2_ for various polarization configurations, providing a platform for future investigations concerning defects in this material.

## Conflicts of interest

The authors declare that they have no competing financial interests.

## Supplementary Material

CP-024-D1CP04322F-s001
