# Supplementary material for: Raman tensor of zinc-phosphide (Zn3P2): from polarization measurements to simulation of Raman spectra
Source: Phys Chem Chem Phys. 2021 Nov 24;24(1):63–72. doi: 10.1039/d1cp04322f (PMC8694062; doi:10.1039/d1cp04322f)
Supplement: CP-024-D1CP04322F-s001 [file CP-024-D1CP04322F-s001.pdf]

Electronic Supplementary Material (ESI) for Physical Chemistry Chemical Physics.
This journal is © the Owner Societies 2021

Supporting Information for:

Raman tensor of zinc-phosphide (Zn_3P_2): from polarization measurements to simulation of Raman spectra

M. Flór,[1] E. Z. Stutz,[1] S. P. Ramanandan,[1] M. Zamani,[1] R. Paul,[1] J-B. Leran,[1] A. P. Litvinchuk,[2] A. Fontcuberta i Morral[1,3], M. Dimitrievska,[1]*

1 - Laboratory of Semiconductor Materials, Institute of Materials, Faculty of Engineering, Ecole Polytechnique Fédérale de Lausanne, 1015 Lausanne, Switzerland.

2 - Texas Center for Superconductivity and Department of Physics, University of Houston, Houston, Texas 77204-5002, United States of America

3 - Institute of Physics, Faculty of Basic Sciences, Ecole Polytechnique Fédérale de Lausanne, 1015 Lausanne, Switzerland.

* e-mail: mirjana.dimitrievska@epfl.ch

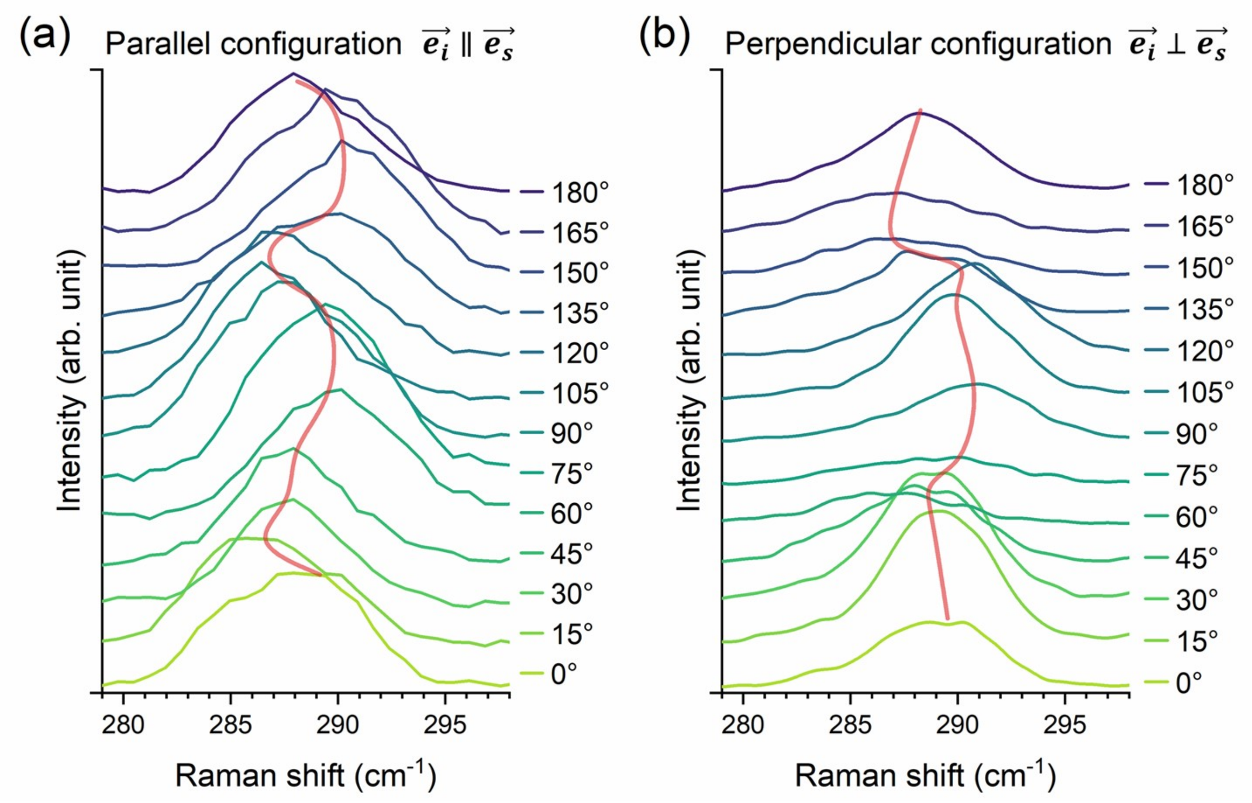

Figure S1. Raman peak at 287 cm^{-1} measured in (a) parallel and (b) perpendicular configuration for various polarization angles showing changes in the peak position and width, and suggesting superposition of two or more vibrational modes.

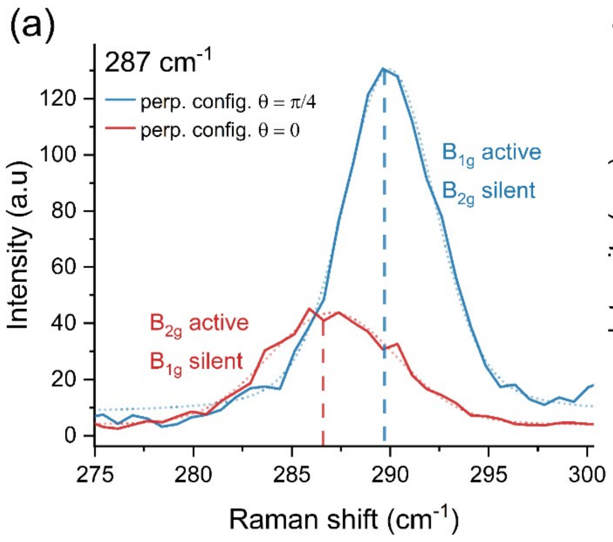

Figure S2. (a) Measured spectrum around the 287 cm^{-1} peak, in two different angular configurations. In red, the B_{1g} mode is cancelled and the B_{2g} mode is active. While in blue, the B_{2g} mode is cancelled and the B_{1g} mode is active. (b) Example of a Raman spectrum deconvolution with Lorentzian curves in perpendicular configuration for polarization angle of $\theta = 22.5^o$, where B_{1g} and B_{2g} modes are simultaneously activated.

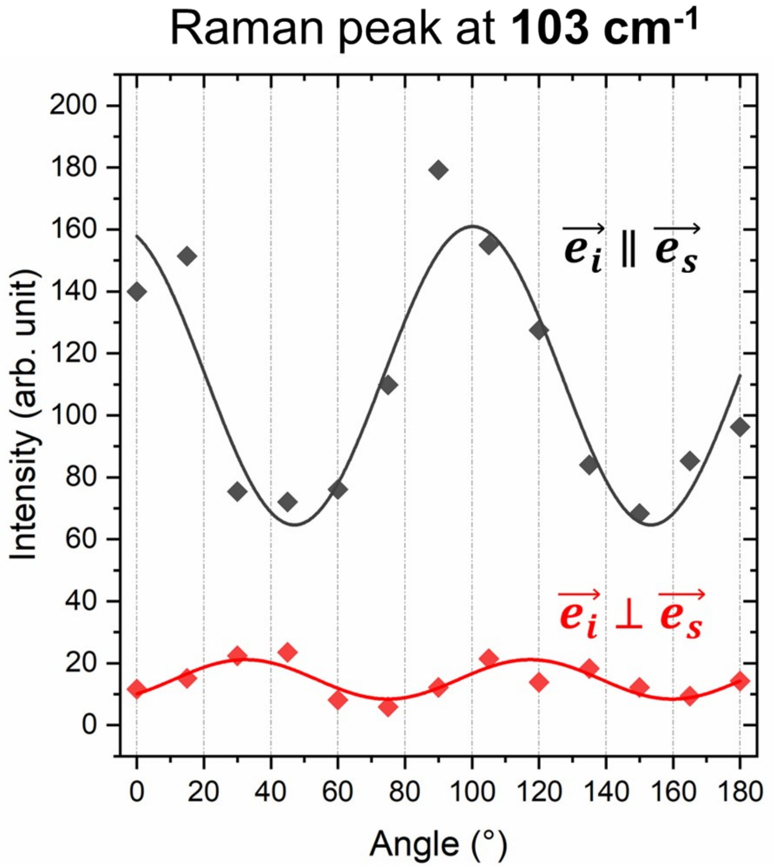

Figure S3. Polarization-dependent Raman intensity of Raman peak centered at 103 cm^{-1}, corresponding to E_g mode.

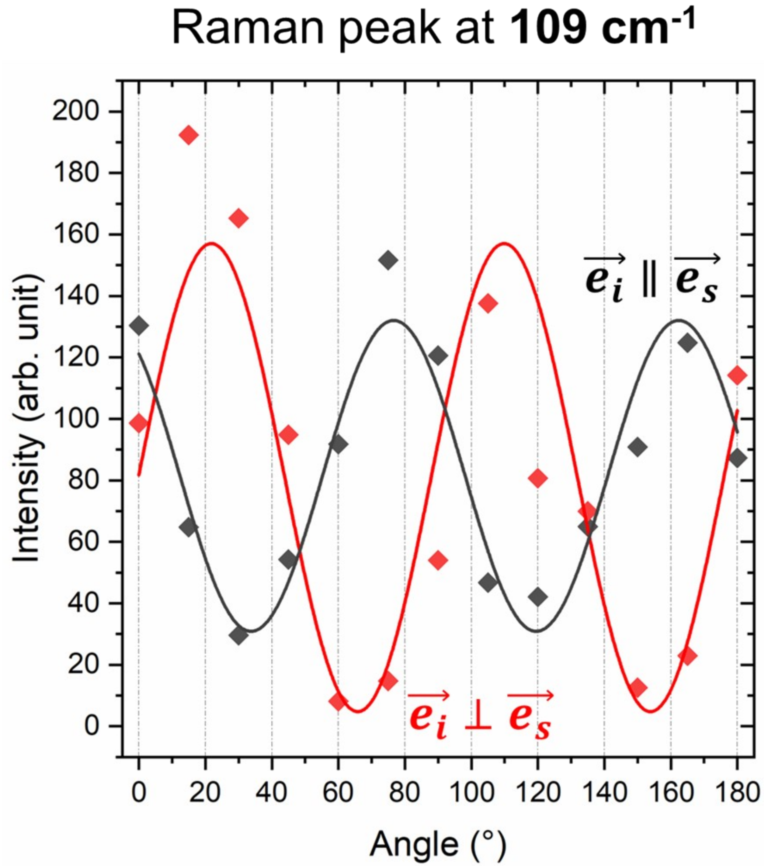

Figure S4. Polarization-dependent Raman intensity of Raman peak centered at 109 cm^{-1}, corresponding to B_{1g} mode.

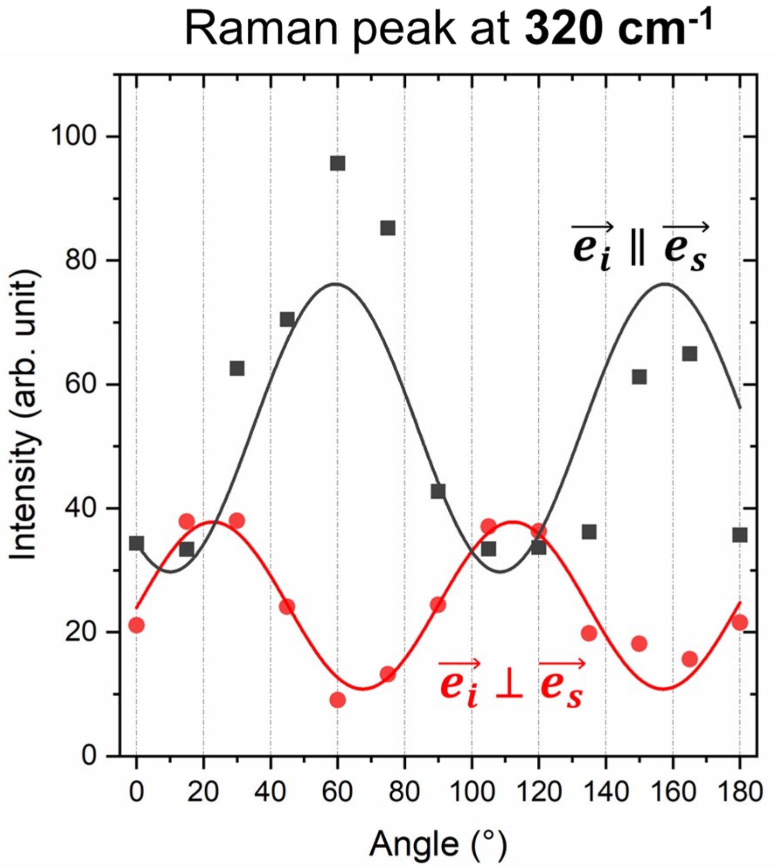

Figure S5. Polarization-dependent Raman intensity of Raman peak centered at 320 cm^{-1}, corresponding to E_g mode.
